# Validation of an effective, low cost, Free/open access 3D-printed stethoscope

**DOI:** 10.1371/journal.pone.0193087

**Published:** 2018-03-14

**Authors:** Alexander Pavlosky, Jennifer Glauche, Spencer Chambers, Mahmoud Al-Alawi, Kliment Yanev, Tarek Loubani

**Affiliations:** 1 Faculty of Medicine, University of Western Ontario, London, Ontario, Canada; 2 No institutional affiliation (Independent contractors), Cologne, Germany; 3 Glia, Inc., London, Canada; 4 Division of Emergency Medicine, Department of Medicine, University of Western Ontario, London, Ontario, Canada; 5 Division of Emergency Medicine, London Health Sciences Centre, London, Ontario, Canada; 6 Division of Emergency Medicine, Al-Shifa Hospital, Gaza City, Occupied Palestinian Territories; University of Illinois at Chicago, UNITED STATES

## Abstract

The modern acoustic stethoscope is a useful clinical tool used to detect subtle, pathological changes in cardiac, pulmonary and vascular sounds. Currently, brand-name stethoscopes are expensive despite limited innovations in design or fabrication in recent decades. Consequently, the high cost of high quality, brand name models serves as a barrier to clinicians practicing in various settings, especially in low- and middle-income countries. In this publication, we describe the design and validation of a low-cost open-access (Free/Libre) 3D-printed stethoscope which is comparable to the Littmann Cardiology III for use in low-access clinics.

## Introduction

Since its introduction in 1819 by René Laennec[1], the acoustic stethoscope has been an integral part of clinical medicine. Despite the lack of major structural design innovations over recent decades, modern stethoscopes can be an expensive part of the physician’s armamentarium, often costing several hundred US dollars. The high cost of modern stethoscopes remains a significant barrier to physicians and allied health professionals practicing in the developed word and in low- and middle-income countries, where few affordable high-quality options exist. Traditionally, the selection of a stethoscope often does not involve the acoustic properties of the model[2] with most users selecting an expensive brand-name stethoscope such as the Littmann Cardiology III or other premium brands. However, previous studies that compared stethoscope brands have concluded that cost does not correlate with better diaphragm sound quality at relevant frequencies, compared with lower-cost alternatives[3,4]. While other 3D printed stethoscope models that would be low-cost to produce can be found online[5–15], we are not aware of any that have been used clinically or research-validated.

Numerous groups have previously attempted to standardize methods to determine the efficacy of acoustic stethoscope models[2,16–20], but currently no accepted standardized modality exists. Consequently, the performance of any acoustic stethoscope is little more than the manufacturer’s claim or the subjective opinion of the user. Some groups have attempted to objectively compare acoustic stethoscope models and currently two competing methods of measuring frequency response exist. The first method uses air coupling to transmit frequencies[2–4,16,17] while the other uses a phantom to simulate vibrations of the chest wall[19,21,22]. These methods allow investigators to quantitatively compare the sensitivity of a stethoscope model compared with another.

In this article, we describe the construction and validation of a low cost, Free/open access 3D printed acoustic stethoscope—referred to here as the ‘Glia model’. The aim of this research is to give low budget health care systems affordable access to an effective stethoscope for a cost under $5 USD and to contribute to the body of literature by ensuring this stethoscope is rigorously tested and released as Free/open access hardware. To achieve this, we utilized 3D printing, a technology that is advancing rapidly and becoming increasingly inexpensive[23]. 3D printing technology has been used by others, especially for work on prosthetics[24–26], splints[27,28] and preoperative planning[29]. It has also been proposed as a partial solution to problems in supply chain management during disasters[30], such as by Global Humanitarian Lab[31] and Field Ready[32]. The flexibility of 3D printing technology also allows users to augment our design to fit their own needs. We also attempt to make our bills of material, construction methodology and validation methods accessible and low cost, allowing others to validate our design independently with ease.

## Methods

### Stethoscope design

**Fig 1:**
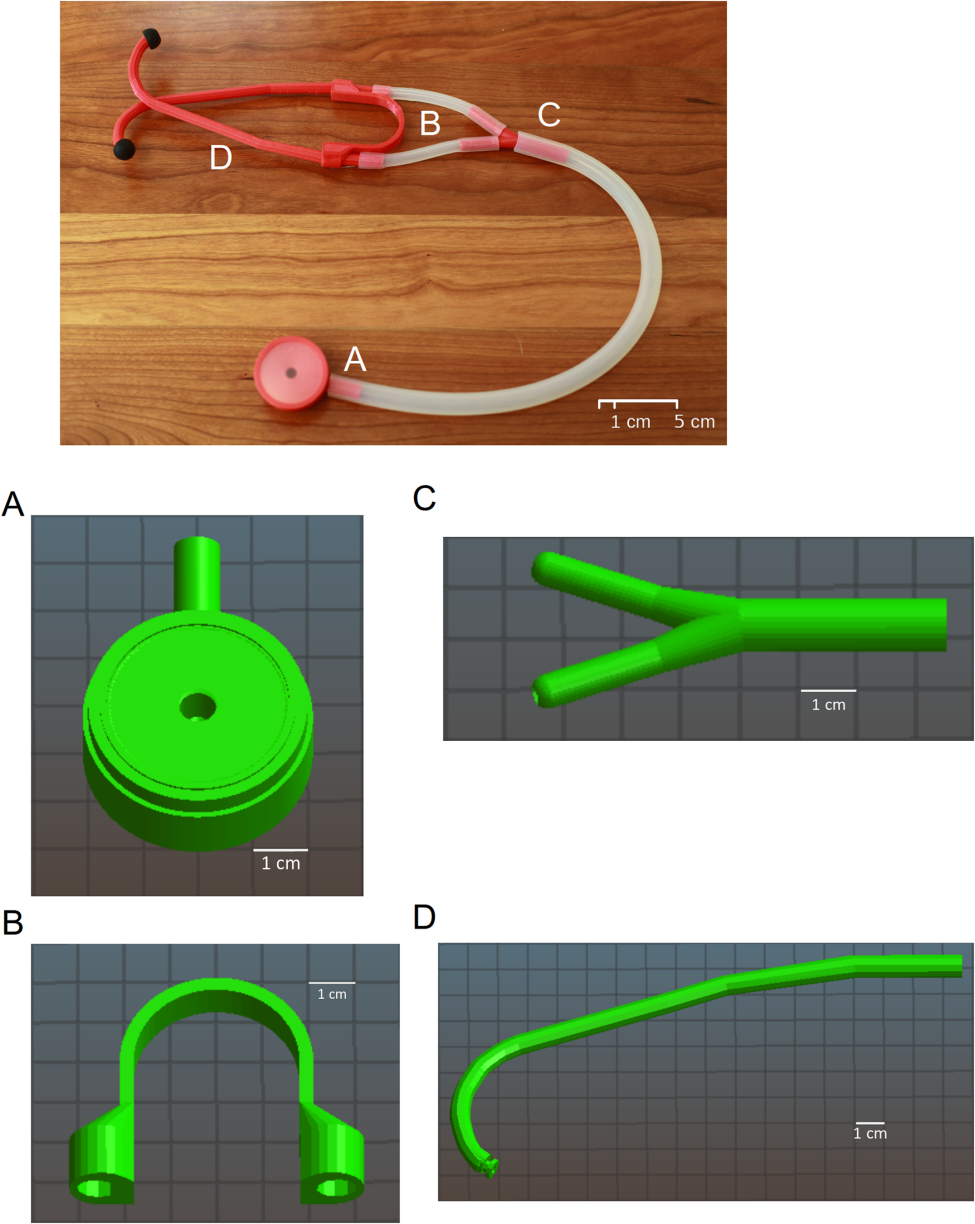
Computer aided design and assembly of the 3D printed stethoscope. Digital models of the 3D printed stethoscope parts are shown in Fig 1A. From left to right: the head, Y piece and ear tube are shown. An earplug mold design is also shown in Fig 1B. Each part was 3D printed in ABS, with the assembled stethoscope is shown in Fig 1C using the bill of materials listed in Table 1.

Design of the Glia model 3D printed stethoscopes was done using Free/Open Source Software (FOSS) so as to keep costs low and allow others easy access to examine and modify code. CrystalSCAD (https://github.com/Joaz/CrystalScad, Germany) was used to create digital models of the stethoscope head, two ear tubes and an ear plug mold due to its ability to create complex shapes in a way that was not possible with OpenSCAD at the time. OpenSCAD (http://openscad.org, Canada) was used to create digital models of the Y-piece, stethoscope ring and spring (Fig 1A). Since its original creation as documented in this paper, the eartubes have been completely ported to OpenSCAD. The stethoscope head is presently a hybrid of CrystalSCAD and OpenSCAD. As the ear plug mold is no longer used in our current production process, its archived version also remains in CrystalSCAD.

Other accessory hardware is required, such as the plastic diaphragm, tubing and ear buds. These are listed in Table 1.

**Table 1:** Bill of materials for the Glia model stethoscope (100% infill).

Item	Dimensions (mm)	Weight (g)	Cost using ABS pellets (USD)	Cost using ABS filament (USD)
Stethoscope head	44.30 x 62.45 x 17.80	23.82	$0.17	$0.57
Stethoscope Y piece	70.89 x 29.94 x 9.00	2.84	$0.02	$0.07
Stethoscope ear tubes	170.79 x 83.62 x 5.80	12.52	$0.09	$0.30
Stethoscope ring	r = 21, h = 7	0.81	$0.01	$0.02
Stethoscope spring	91.25 x 111.62 x 15.05	8.05	$0.06	$0.19
Silicone tubing	L = 400–12 OD, 8 ID 2 x L = 90–6 OD, 4 ID		$1.93	$1.93
Diaphragm	r = 20		$0.06	$0.06
Silicone earbuds	n/a		$0.02	$0.02
Electricity usage	0.5 KWh at mid-peak rates		$0.04	$0.04
Total			$2.40	$3.20

All print designs can be downloaded for free at https://github.com/GliaX

### Stethoscope printing and assembly

Each part was printed on a commodity 3D printer (Prusa Mk II, 1.75 mm filament diameter, 0.4 mm nozzle diameter, no scaffolding or support) using acrylonitrile butadiene styrene (ABS) with 100% infill as indicated and 0.2 mm layer height. A 40 cm silicone 12 mm outer diameter (OD), 8 mm inner diameter (ID) tube was attached between the stethoscope head and the larger bore of the Y piece. Two 9 cm silicone 6 mm OD, 4 mm ID tubes were attached between the smaller bore of the Y piece and the ear tubes. A diaphragm was cut from a Staples brand PVC report cover (Swing-lock report cover, clear with black spine; UPC 718103160223) by turning a sharp caliper and creating a circular diaphragm with a 40 mm diameter. This diaphragm was attached to the stethoscope head with a printed ABS ring. A printed ABS truss that has some spring properties by design is used as a spring for the ear tubes.

The earbuds are generic earbuds from commodity earbud-style headphones that are widely available and of negligible cost. The final construction of the current model can be seen in Fig 1C.

Costs in Table 1 were calculated using the density of PA757 ABS (1.05 g/cm^3^)[33] and the part weight, which is calculated using version 1.3.0-dev of the Slic3r software (https://slic3r.org, Italy). We assumed the price of 12 lbs (5.4545 kg) of virgin PA757 ABS pellets to be $38.99 USD[34]. Commercial ABS filament was assumed to cost $23.96 USD/kg[35]. Electricity costs were estimated using the Ontario Energy Board’s current mid-peak rates[36,37] multiplied by the printer’s expected electricity use based on measurements of a similar printer with similar temperatures[38] printing for four hours then multiplied by 0.8 for an approximate conversion to US currency. This number was rounded up to the nearest cent. The energy costs of filament extrusion are negligible when estimated based on previous reporting[39] and were thus not included.

### Acoustic transfer

The frequency response of Glia model stethoscopes, compared with the Littmann Cardiology III, was determined using an experimental setup modeled from a phantom-based frequency response setup previously described[19]. A latex balloon filled with 2 L (2000 g) of water was used as a phantom and each stethoscope was applied to the surface by hand. Phantom excitations were supplied by an external vibrating speaker which was placed in contact with the balloon and sound was played at 86 Hz intervals between 0 and 5000 Hz (white noise) for 15 seconds. The output of each stethoscope was recorded by a microphone which was placed in a silicon tube attached to the stethoscope head for spectral analysis. Spectral analyses such as these have been used successfully in the past to analyze breath sounds recorded from individuals with lung pathology[40]. The simplicity of this design was intended to allow other users to validate our design independently.

## Results

After many iterations, we successfully designed a working stethoscope, known as the Glia model (Fig 1), at a total cost of $2.83 USD using recycled ABS pellets. A bill of materials and cost breakdown can be found in Table 1.

**Fig 2:**
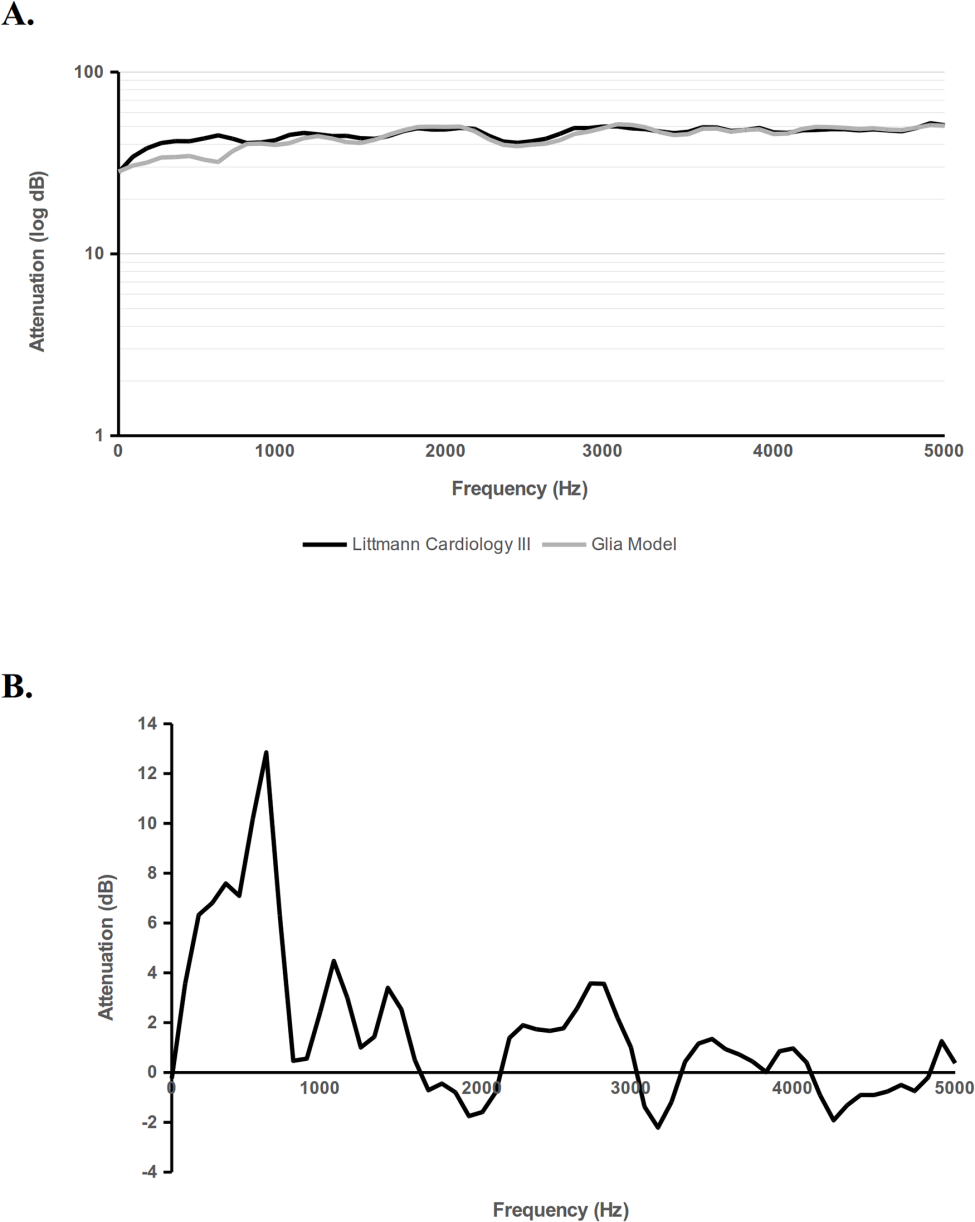
Calibration and comparison of 3D printed Glia model stethoscopes to the Littmann Cardiology III. Stethoscope output responses were measured using the equipment setup described in the methods. Each stethoscope model recorded input sound at multiple frequencies and the change in amplitude between input and recorded sound was documented (lower log attenuation is better) for each stethoscope (Fig 2A). The decibel difference in attenuation (Glia minus Littmann) is shown across all frequencies tested where values above 0 dB indicate the Glia model attenuated less sound (Fig 2B).

We compared the Glia stethoscope to the Littmann Cardiology III using a phantom, as described in the methods. At all frequencies tested, the Glia model performed similarly to the Cardiology III (Fig 2A). The difference in attenuation (dB) of the Glia model to the Littmann Cardiology III is shown in Fig 2B with values greater than 0 dB indicating that the Glia attenuated less sound.

## Discussion

Using a phantom-based method, we show here that the Glia model stethoscope, at a cost of $2.40 USD, is comparable to the Littmann Cardiology III across a range of spectral frequencies from 86 Hz to 5000 Hz, making it a low-cost, suitable alternative to those who cannot access or afford a high-cost model.

The stethoscope is one of the most widely used instruments in modern medicine, allowing clinicians to detect subtle changes in heart, lung and vascular sounds. Despite a lack of major innovation in design or fabrication since Dr. Littmann patented his stethoscope in 1963, the acoustic stethoscope remains an expensive piece of equipment that creates a cost barrier for physicians practicing in low- and middle-income countires. This study aims to create a validated high quality acoustic stethoscope at a cost under $5 USD.

The quality and intensity of the sound reaching the earpiece from the diaphragm is dependent on nearly every piece of the stethoscope as well as the physiology of the user. These variables have been previously summarized[3] and include the size and volume of the bell[18,41]; hardness of the inner cavity of the bell[42]; improperly fitted components allowing air leaks and loss of sound[41]; the thickness, size and tautness of the diaphragm and the interior smoothness, rigidity, length and diameter of the tubing[43,44]. Additional user related factors include improperly fitted ear pieces that allow air exchange[4,41,44,45]; anatomical variations of the auditory canal of the user[45]; background noise[46] and training[47]. Many of these variables needed to be considered when designing the Glia model stethoscope, particularly physical properties such as channel diameter through the 3D printed parts and infill percentage, which ultimately determines the density and hardness of the parts. We also tried several printing materials including poly-lactic acid (PLA) and ABS. Of particular challenge was creating the interface between the ear tube and the spring to prevent rotation when the ear tubes were pulled apart.

As previously mentioned, no standard method of determining the acoustic response of stethoscope models currently exists. Previous studies have attempted to objectively quantify stethoscope efficacy[19,21,22,41] and previous comparisons between brands indicate that no significant correlation between cost and quality exists[3,4]. However, there may be some subjective decrease in efficacy when using low-quality disposable stethoscopes[47].

The Littmann Cardiology III has recently become unavailable. Its replacement, the Littmann Cardiology IV costs $190 USD[48], which is comparable to other brand name stethoscopes such as the Welch Allyn Harvey Elite ($150 USD[49]). Ultimately, however, the usefulness of any stethoscope is dependent on user preference and so we encourage those with access to a 3D printer to build and test our model independently. The protocol listed in the methods has been purposely designed to be replicable using commonly available materials. Any printer capable of printing in ABS should be able to create our device, including RepRap printer designs used by our group[50]. Printers of sufficient quality and reliability can be easily obtained or built internationally for less than $1,000 USD[51].

The Glia model stethoscope is a class I medical device according to Health Canada and the FDA. In Canada, a non-profit company was incorporated to manufacture stethoscopes and has received a Medical Device Establishment Licence from Health Canada. The stethoscope is in clinical use in London, Canada at the London Health Sciences Centre. It has also been trialed and was gradually introduced in the Gaza strip, an area with extremely limited access to medical devices. Hospitals in Gaza are self-sufficient producers of these stethoscopes.

### Low cost does not mean low quality

This work aimed to produce a stethoscope of comparable quality to those premium-brand devices unobtainable due to economic, political or military factors. We believe this aim was achieved as shown in the results above. This work is significant in both developed and low- and middle-income countries (LMICs) as it introduces two cost-saving innovations: the use of plastic and 3D printing to create a product equal to metal alternatives and the dissemination of plans and bills of material through a Free and open source license.

Our results suggest that the use of inexpensive techniques to produce medical devices does not necessitate the lowering of quality standards. This presents a challenge to an implicit assumption found in some literature and organizations that lower standards may be necessary for the developing world[52,53]. An example pertinent to 3D printing is the use of 3D printed upper limb prosthetics based on the e-Nable project[26] in LMICs. These prosthetics have been found to be inadequate in many important activities as per a standardized assessment[54]. Despite this, the implicit argument is that poor quality devices are acceptable because of the present lack of access to devices[53].

### Future research

This project was the first of several planned open access medical device projects. Future plans include expanding access by providing validated models of other pieces of medical equipment, including pulse oximeters, tourniquets, otoscopes and ECG machines, allowing health institutions to sustainably produce affordable, high quality equipment for many clinicians.
